# Hailey-Hailey Disease Successfully Treated With Adalimumab: A Case Series

**DOI:** 10.7759/cureus.67227

**Published:** 2024-08-19

**Authors:** Abdiel J Alicea-Negrón, Reina M González-Barreto, Jose R González-Chávez

**Affiliations:** 1 Medical School, University of Puerto Rico School of Medicine, San Juan, PRI; 2 Dermatology, Ponce Health Sciences University, Ponce, PRI

**Keywords:** case series, tnf alpha, adalimumab, genodermatosis, hailey-hailey disease

## Abstract

Hailey-Hailey disease is an autosomal dominant disorder caused by a mutation in the ATP2C1 gene and characterized by recurrent blisters, erosions, and crust in intertriginous areas. Currently, there are no curative treatments for Hailey-Hailey disease, and therapeutic strategies are focused on controlling skin microbial colonization, infection, and inflammation. Recent efforts have aimed to find therapies that target the biochemical pathway involved in the pathogenesis of this disease. Several case reports indicate the use of different biological agents to achieve long-term remission in patients with recalcitrant Hailey-Hailey disease. Tumor necrosis factor-alpha inhibitors have been used to treat and maintain remission in recalcitrant Hailey-Hailey disease patients, but additional reporting and studies are required. In this case series, we report three cases of recalcitrant Hailey-Hailey disease whose lesions were successfully controlled with adalimumab.

## Introduction

Hailey-Hailey disease (HHD), also known as benign familial pemphigus, is a rare acantholytic autosomal dominant disorder characterized by the development of relapsing and recurrent blisters, erosions, and crust in intertriginous areas. HHD is caused by a mutation in the ATP2C1 gene, which encodes for the human secretory pathway Ca2+/Mn2+-ATPase isoform 1 (hSPCA1) of the Golgi apparatus [1]. This pump supplies the Golgi lumen with Ca2+ and Mn2+ and helps to maintain their normal intracellular concentrations. Mutations in this gene are responsible for a decrease in Golgi calcium storage, calcium-dependent translational modifications, and calcium-dependent protein sorting and trafficking, leading to the characteristic acantholytic pattern in this disease [1]. There is no specific therapy for HHD, and the therapeutic approach involves managing exacerbating factors, secondary infections, and skin inflammation. These therapies include topical and oral corticosteroids, antimicrobials, topical calcineurin, zinc paste, phototherapy, botulinum toxin injections, and lasers. Emerging therapies focus on targeting specific molecules that may be involved in the inflammatory pathway of this disease. These medications include naltrexone, apremilast, dupilumab, and tumor necrosis factor (TNF)-α inhibitors [2-8]. Two reports have documented successful treatment of severe HHD after the administration of a TNF-alpha inhibitor [7,8]. TNF-α inhibitors, such as adalimumab, downregulate the inflammatory response mediated by TNF-α by binding to the TNF-α receptor in human cells. By doing this, cellular processes that are directly or indirectly dependent on TNF-α are affected [9]. However, additional reports and long-term data are still lacking. Given the disease’s chronic course and lack of specific treatments, patients may encounter psychological distress and impaired quality of life (QOL) [10]. Here, we report a case series of three patients with recalcitrant HHD who were successfully treated with adalimumab, a TNF-α monoclonal antibody.

The patient in Case 1 was previously presented as a poster at the American College of Physicians Puerto Rico Chapter 2023 Clinical Vignettes & Research Abstracts on November 18, 2023, and at the Latin Medical Student Association RCM 2024 Research Symposium on April 20, 2024. The case series was presented as an oral presentation at the 'Sociedad Dermatologica de Puerto Rico' (Dermatological Society of Puerto Rico) 2024 Annual Convention on June 28, 2024.

## Case presentation

Case 1

A 43-year-old man presented to the dermatology clinic with a six-year history of crusted, scaly, erythematous plaques that appeared intermittently on various parts of his body. At the time of the visit, he had lesions in the axilla and mid-lower back, which were associated with pain and a foul-smelling discharge. The diagnosis of HHD was confirmed through biopsy and clinical evaluation. Despite receiving a series of therapies over the years to control the condition, the treatments provided only slight improvement, leading the patient to return with persistent symptoms.

**Figure 1:**
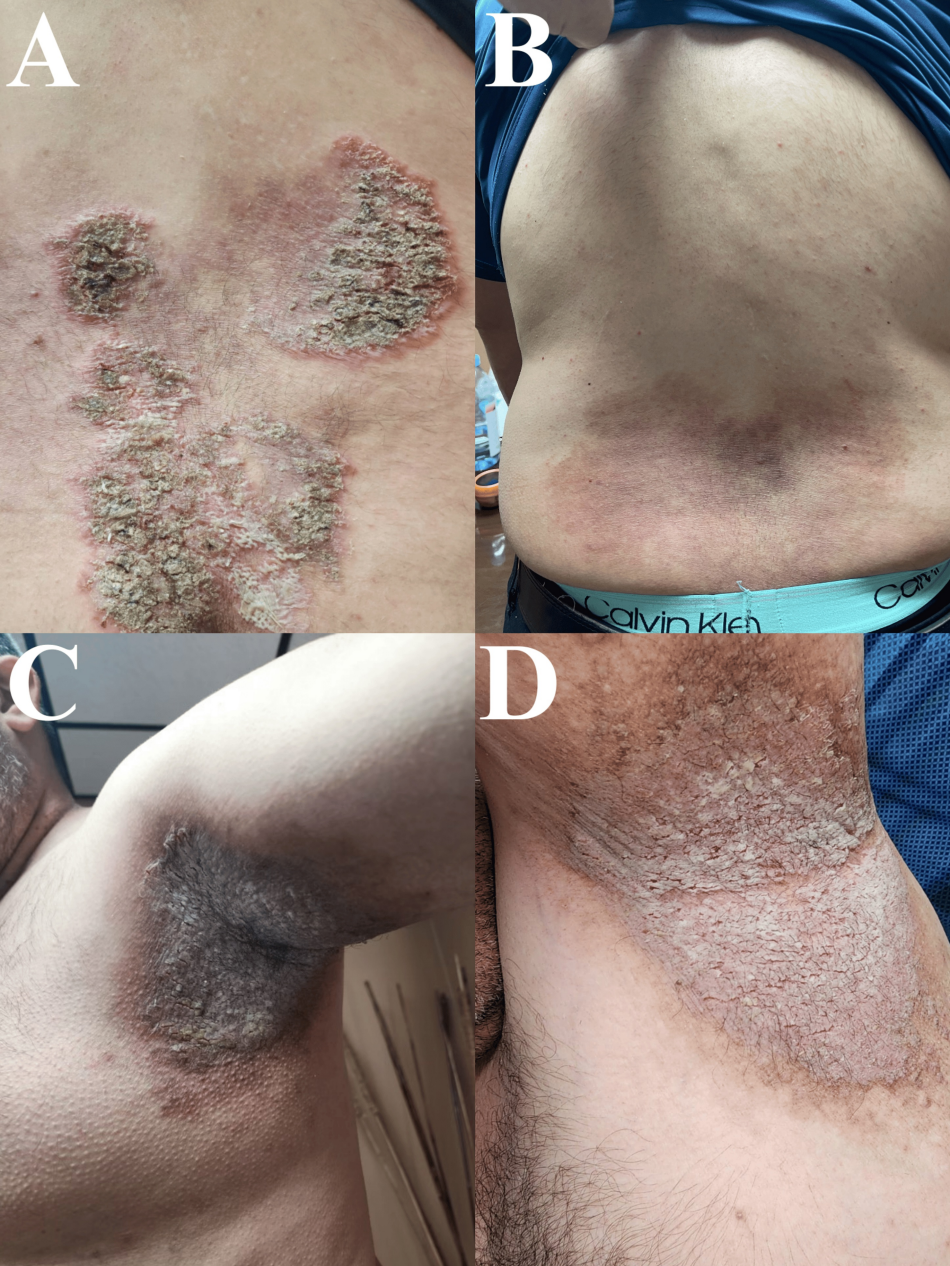
Hailey-Hailey disease lesions before and after treatment with adalimumab. A, C: initial erythematous, crusted, and erosive Hailey-Hailey Disease lesion before starting adalimumab; B, D: lesions after two doses of adalimumab

Adalimumab was offered to the patient as an off-label treatment, which he agreed to initiate as soon as possible. Adalimumab was initiated with a subcutaneous induction dose of 80 mg, followed by a subcutaneous maintenance dose of 40 mg every 14 days. Following the induction dose, an 80-100% improvement was observed within the first two doses (Figure 1). 

**Figure 2:**
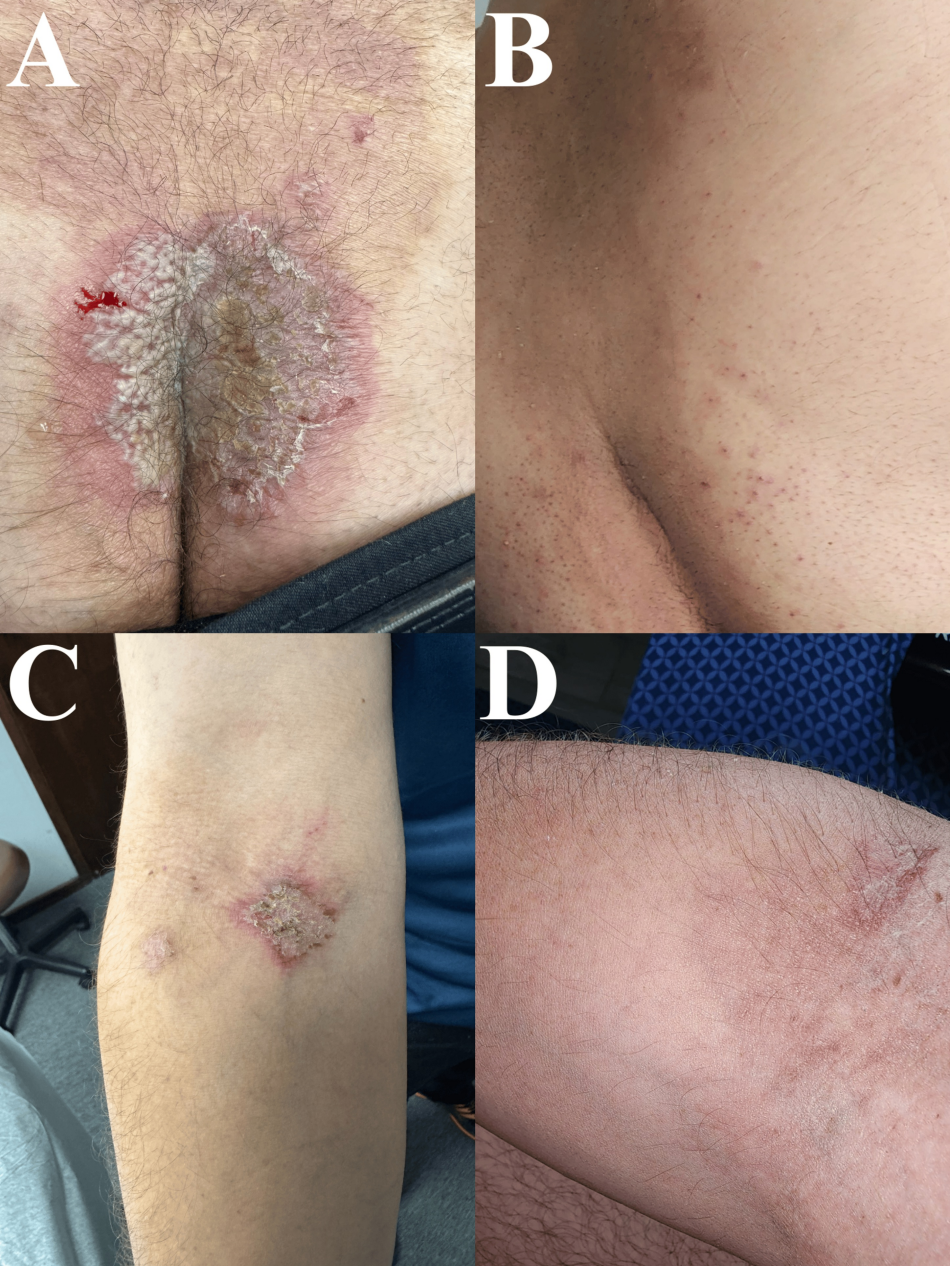
Before and after pictures of the patient in Case 1, reinitiated on adalimumab. Recurrence of Hailey-Hailey disease lesions in the superior intergluteal cleft (A) and right antecubital fossa (C) before (A and C) and after (B and D) four doses of adalimumab.

**Figure 3:**
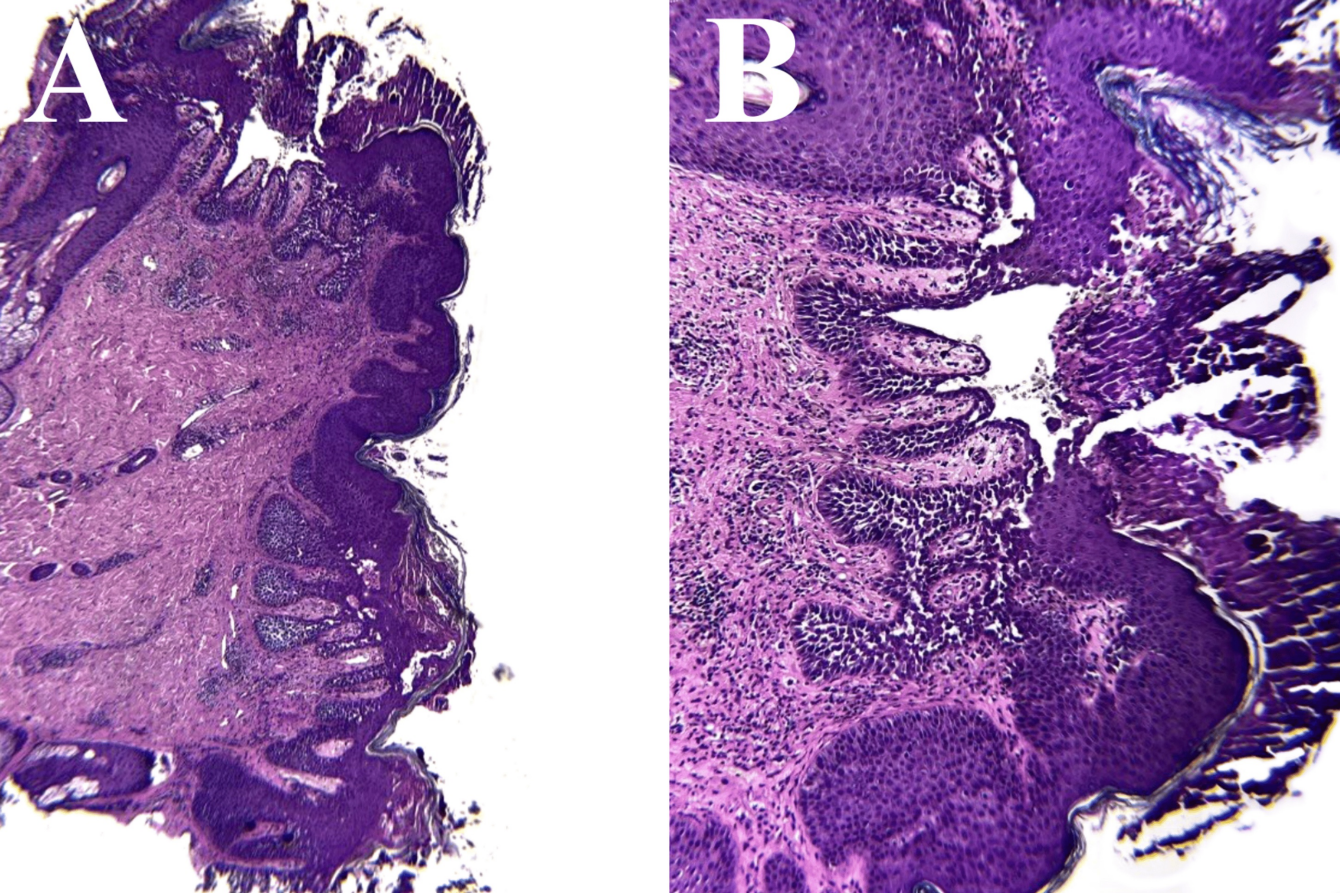
Hematoxylin and Eosin (H&E) staining at two different magnifications, 100x (A), 200x (B). Both images depict the characteristic pattern of intraepidermal vesicles with dilapidated brick appearance of Hailey-Hailey disease.

The patient sustained remission for six months until the discontinuation of therapy due to a lack of insurance coverage. The patient reported being completely clear of HHD lesions for approximately three months, after which time the lesions reoccurred. Four months after the recurrence of the HHD lesions, the patient presented to our clinic. Physical examination revealed erythematous, crusty, and suppurative plaques localized on the upper intergluteal cleft and right antecubital fossa (Figure 2A, 2C). These lesions were notably less severe than the previous lesions. A skin biopsy was taken to confirm the recurrence of HHD (Figure 3). A dose of 40 mg of adalimumab was administered, followed by a maintenance dose every 14 days. An assessment after four doses noted a substantial improvement in the lesions (Figure 2B, 2D).

Case 2

A 51-year-old woman with a 20-year history of clinical and biopsy-confirmed Hailey-Hailey disease was offered adalimumab as a potential treatment for the disease. Throughout the 20 years, the patient had HHD lesions in the axilla, inguinal folds, and below the nail of the right hallux, for which she tried multiple treatment options, but none of them achieved substantial remission. Symptoms reported by the patient include heat intolerance, excess sweating, clear discharge in lesions, cervical and inguinal lymphadenopathy, malodor, sexual dysfunction, and stress, all of which significantly impact her QOL.

**Figure 4:**
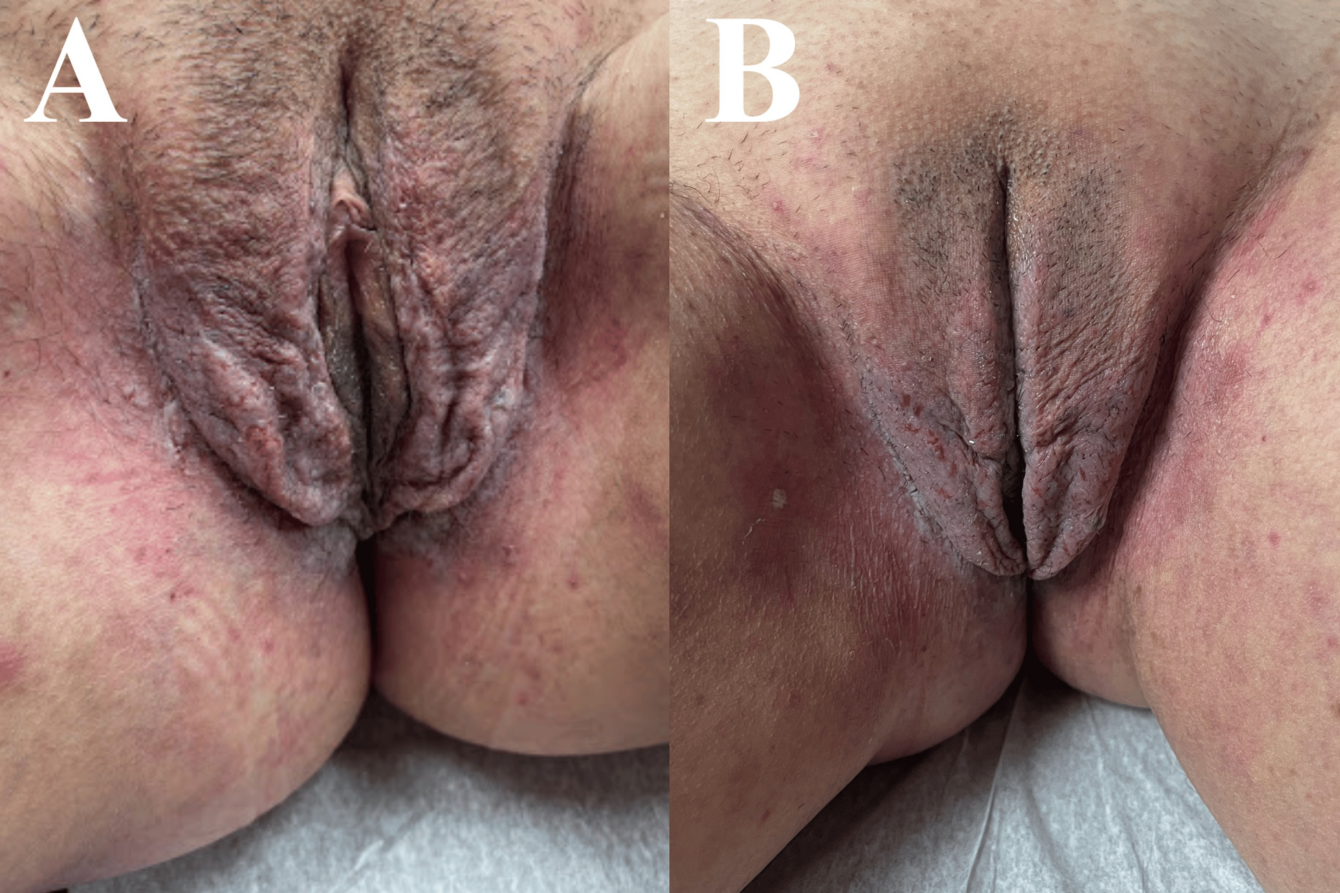
Before and after images of the vulvar Hailey-Hailey disease lesions of the patient in Case 2. A: Hailey-Hailey disease lesions in the vulva and intertriginous area before the initiation of adalimumab; B: Hailey-Hailey disease lesions before receiving her third dose, showing a diminution in inflammation.

At the time of the visit, the patient exhibited lesions on the vulva. Associated symptoms included malodor, dyspareunia, sexual dysfunction, and dysuria. The use of adalimumab as an off-label medication for HHD was discussed, and the patient agreed to try it. She was initiated on a subcutaneous induction dose of 80 mg, followed by 40 mg every 14 days. Following the first 30 days, at a follow-up visit to receive the third dose, the patient had >60% improvement in the lesions initially presented (Figure 4). After the fourth dose, the patient’s prescription was adjusted to one monthly dosage.

**Figure 5:**
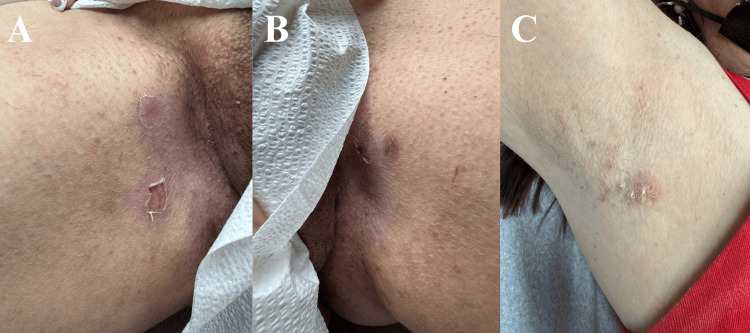
Acute Hailey-Hailey disease lesions that appear days before the monthly scheduled maintenance dose in the right (A) and left (B) medial thigh and right axillary (C) regions.

To date, the patient has received six doses of adalimumab, and the results are exceptional. Besides the occasional flares when it is almost time to receive her monthly maintenance dose (Figure 5), the patient reports an improved QOL and a decrease in associated symptoms.

Case 3

**Figure 6:**
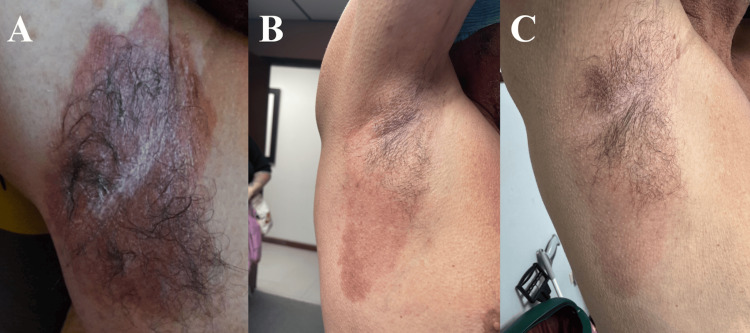
Axillary Hailey-Hailey disease lesions of the patient in Case 3 before and after adalimumab. A: Hailey-Hailey disease lesion in the left axilla before initiation of adalimumab. The same lesion after two (B) and three (C) doses of adalimumab.

A 64-year-old man (uncle to the woman in Case 2) with a 37-year history of biopsy-confirmed HHD was treated with adalimumab for his Hailey-Hailey disease lesions on the left axilla (Figure 6A). After trying almost every treatment available for HHD, the patient agreed to initiate a TNF-α inhibitor trial. The patient received an initial subcutaneous dose of 80 mg adalimumab, followed by two subcutaneous maintenance doses of 40 mg every 14 days and then every 30 days. After the first two doses (Figure 6B), a significant improvement of over 95% was observed in the lesions of the axilla (Figure 6C).

**Figure 7:**
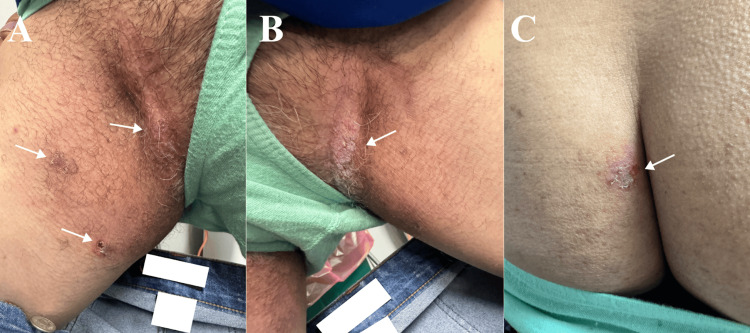
Acute Hailey-Hailey disease lesions that appear days before the scheduled adalimumab maintenance dose. White arrows showing typical Hailey-Hailey disease lesions at the right (A) and left (B) inguinal folds and left intergluteal cleft (C).

To date, the patient reports experiencing acute flares only when it is time for his monthly maintenance dose, which resolve with the administration of the medication (Figure 7).

## Discussion

We report three cases of patients with recalcitrant HHD who were treated with adalimumab. All three cases showed more than an 80% improvement in their lesions and reported an improvement in their QOL.

The patient in Case 1 exhibited a favorable response to the lesions located on the lower back each time he was treated with adalimumab. He also experienced a favorable response in the lesions at the antecubital area, but he developed acute lesions in the same area days before his maintenance dose (Figure 2D). Unfortunately, the lesions in the axilla only showed mild improvement. However, the patient reports diminished pain, which significantly improves his QOL and allows him to continue his daily activities without any limitations.

Cases 2 and 3 reported induration and tenderness in their skin, followed by an acute HHD flare as the maintenance dose date approached. However, these lesions resolved after the administration of adalimumab.

To date, the role of TNF-α in the pathophysiology of HHD has not been fully described. The pathophysiology of HHD entails the downregulation of hSPA1C, leading to the disruption of calcium homeostasis within epidermal cells. It is well documented that the epidermal calcium gradient, primarily found in the endoplasmic reticulum and the Golgi apparatus [11], is an essential factor in keratinocyte differentiation and the epidermal barrier. Disruption in calcium concentrations throughout the epidermis results in disturbance of the processing, translocation, and stability of junctional proteins, inducing acantholysis, and compromising the skin permeability barrier [1]. Recent studies have shown impaired actin reorganization and decreased cellular ATP levels in HHD keratinocytes [12]. An impaired calcium pump into the Golgi apparatus results in increased intracellular calcium concentration despite having a low calcium content in basal cells. This, in turn, stimulates keratinocytes to increase gene expression for differentiation markers and desmosome formations [13]. However, in HHD patients, this process is impaired, creating a detrimental cycle of increased desmosome formation signals and impaired synthesis. Increased TNF-α levels have been observed in keratinocytes after the disruption of the epidermal barrier [14]. Also, TNF-α has been shown to increase cytosolic calcium concentration in various cell types [15,16] and modulate local inflammatory responses through calcium homeostasis [11], which may further exacerbate the pathogenesis of HHD. 

Recent evidence suggests that TNF-α and interleukin-6 (IL-6) enhance the growth of herpes simplex virus-1 in differentiated keratinocytes, leading to delayed skin healing. Additionally, mRNA levels of ATP2C1 decrease when human keratinocytes are stimulated with IL-6 [17], a cytokine-induced by TNF-α through the NF-κB pathway [18]. Recent case reports highlight the use of apremilast as a treatment for HHD, attributing its effects to the downregulation of CXCL10 and consequent modulation of the TH1 response [3]. The downregulation of CXCL10 decreases proinflammatory cytokines, including TNF-α [19,20]. Another drug that has been used to treat recalcitrant HHD is naltrexone, a toll-like receptor 4 antagonist that leads to a decrease in TNF-α, IL-6, and nitric oxide [2].

## Conclusions

Although further investigation is required, TNF-α is notably involved in the pathophysiology of HHD, either by inducing alterations in calcium homeostasis within the epidermis or by stimulating proinflammatory cytokines to exacerbate symptoms and complications. This case series provides evidence and plausible explanations for how TNF-α inhibitors may contribute to achieving long-term remission in patients with recalcitrant HHD. The reasons why our patients responded differently and why acute HHD lesions appeared while using the medication are not completely understood and require further investigation. It is important to emphasize that all three patients in this case series reported a drastic improvement in their quality of life while using adalimumab. Our goal with this case series is to contribute to the limited literature supporting the use of TNF-α inhibitors in the treatment of HHD. This may encourage future research efforts to understand the role of TNF-α in HHD, as well as to develop randomized trials to assess the efficacy of adalimumab in this genodermatosis.
